# Contralateral suppression of otoacoustic emission in patients with tinnitus

**DOI:** 10.1016/S1808-8694(15)30059-8

**Published:** 2015-10-19

**Authors:** Mariana Lopes Fávero, Tanit Ganz Sanchez, Ricardo Ferreira Bento, Andreia Ferreira Nascimento

**Affiliations:** a PhD in Otolaryngology - FMUSP, Otolaryngologist at the HSPM and DERDIC/PUCSP.; b Associate Professor of Otolaryngology - FMUSP.; c Associate Professor of Otolaryngology - FMUSP.; d PhD., Professor at the Department of Preventive Medicine - FMUSP.

**Keywords:** Tinnitus, Efferent pathways, Laterality, otoacoustics emissions

## Abstract

**Introduction:** The medial olivocochlear bundle effect is studied through the suppression of otoacoustic emissions and seems to be influenced by the laterality of the central nervous system, presenting no symmetry between right and left ear. A dysfunction of this bundle may be involved in the generation of tinnitus, although this fact was not confirmed. **Objectives:** Study the suppression of distortion product otoacoustic emissions in tinnitus patients. **Material and Method:** A case-controlled study involving 44 tinnitus patients from the Tinnitus Group of the ENT Department of the University of São Paulo Medical School and 44 controls who underwent distortion product otoacoustic emissions testing with and without contralateral noise. Only the results from the right ears from both groups were compared. **Results:** There was a relationship between the presence of tinnitus and the absence of suppression at all frequencies studied (OR>2.1). **Conclusion:** There was a correlation between diminished effectiveness of the medial olivocochlear bundle and the presence of tinnitus.

## INTRODUCTION

Many theories have been conjured up in recent years in an attempt to explain the tinnitus origin. One of them is related to a dysfunction in the efferent auditory system, more specifically the medial olivocochlear tract, as a triggering or maintenance factor of such symptom^1^.

The medial olivocochlear tract acts on the movement of the outer hair cells (OHC) causing their hyperpolarization through the release of acetylcholine in the synaptic slit^2^, ^3^. This hyperpolarization occurs in opposition to depolarization, naturally induced by sound stimulus and keeps the basilar membrane in a proper position for a reliable transduction of the auditory stimulus characteristics^4^.

According to many authors, hyperpolarization is seen clearly by the reduction in the otoacoustic emissions (OAE) amplitude through the use of an acoustic stimulation in the contralateral ear^5^, ^6^, and it is under the influence of the central nervous system, following the hemisphere predominance patterns^7^, ^8^, thus, not presenting similar results between right and left ears in right-handed individuals.

A dysfunction of the efferent system could lead to a loss in outer hair cells modulation, that is, this balance between hyperpolarization and depolarization, generating an abnormal and exaggerated electrical activity that may be misinterpreted as a sound by the Central Nervous System1. This modulation alteration may occur due to a specific lesion in the outer hair cells, causing a reduction in inhibitory efferent stimuli^9^, or by an intrinsic balance alteration between the excitatory and inhibitory components, the former prevailing^10^.

As evidence of this dysfunction, there are reports of tinnitus patients with reductions or total absence of OAE suppression during contralateral acoustic stimulus^11^, ^12^. Notwithstanding, other studies have not yet reproduced these results^13^, ^14^. None of these papers consider the laterality of the nervous system as a variable.

Considering this debate, we designed this study in order to assess Distortion Product Otoacoustic Emissions (DPOAE) with contralateral acoustic stimuli in patients with tinnitus and compare it to the same suppression in normal hearing individuals.

## MATERIALS AND METHODS

This project has been approved by the Ethics Committee of the University Hospital - Medical School of the University of São Paulo (CAPPesq, protocol # 544/00).

A case-controlled study was carried out with 88 participants, further broken down in two groups:

1. Tinnitus Group (TG), made up of 44 individuals with tinnitus regularly enrolled in the Tinnitus Ward - Otolaryngology Department - University Hospital - University of São Paulo. The inclusion criteria were:
  a. constant bilateral tinnitus;
  b. right-handed individuals were selected through the abridged version of the Edinburgh Inventory 15 with maximum age of 60 years;
  c. normal bilateral tonal audiometry (thresholds ≤ 25 dB HL up to 8,000Hz), normal immitanciometry and DPOAE present between 1,000 and 6,000Hz.
2. Control Group (CG), Made up of 44 persons without tinnitus, following the same inclusion criteria used for the TG, except for the tinnitus itself.

The groups were paired by age (TG: 46.7 years, standard deviation ±9.3 years; CG: 46.8 years, standard deviation ± 9.5 years; p=0.98) and gender (14 individuals - 31.8% - were males in each group) and did not present statistical differences as far as tonal audiometry and DPOAE thresholds are concerned (p ≥0.31 and p≥ 0.11 respectively, for all frequencies studied).

In order to measure DPOAE, we used a 503 Celesta cochlear analyzer (Version 3.xx) (Madsen Electronics, Taastrup, Denmark). In order to gather the distortion products (2F1-F2), we used to pure tones at the F2/F1 =1.22 ratio, presented at the level of 70 dBSPL, following the F1 and F2 geometric mean through the DP-gram or amplitude x frequence graph. The suppressive acoustic stimulus used was a white noise generated by a MA 32 model Maico audiometer with a TDH39 headphone and MX 41 pad, at 50 dBHL. In order to avoid DPOAE probe manipulation, the headphone was attached to the contralateral ear before the test onset. Notwithstanding, the OAE probe was systematically tested before signal acquisition in each series.

A 6dB sound/level ration was considered in each frequence instead of total DPOAE amplitude. DPOAE acquisition was carried out first in the absence and later in the presence of white noise in the contralateral ear.

The DPOAE suppressor effect calculation was carried out by subtracting the signal/noise ratio acquired without the contralateral noise from the value of the noise/signal ration with contralateral noise for each specific frequence. Positive values indicated DPOAE suppression, and negative values or zero indicated non-suppression.

In order to measure the link between tinnitus and suppression absence in DPOAE we compared the results between the right ears of tinnitus patients with those from controls, calculating the odds ratio (OR) for each frequency studied, as well as the respective confidence intervals (CI95%).

The link was testes with the chi-squared and the McNemar tests according to the aforementioned methods^16^. We considered p ≤ 0.05 as the level of statistical significance.

## RESULTS

Table 1 shows the results found between the tinnitus patients and the controls in the frequencies studied.

**Table 1:** DPOAE suppression ratio in individuals with and without tinnitus.

Frequency	With Tinnitus	Without Tinnitus	OR	IC_95%_	P
1000 Hz					0,09

Suppression	15(34,1%)	36 (81,8%)	1,0		
No Suppression	29 (65,9%)	8(18,2%)	2,4	0,8 a 6,8	

1500 Hz					< 0,001

Suppression	21 (47,7%)	37 (84,1%)	1,0		
No Suppression	23 (52,3%)	7(15,9%)	15,0	2,0 a 113,6	

2000 Hz					0,001

Suppression	17(38,6%)	41 (93,2%)	1,0		
No Suppression	27(61,4%)	3 (6,8%)	8,0	1,8 a 24,8	

3000 Hz					0,001

Suppression	13(29,6%)	43 (97,7%)	1,0		
No Suppression	31 (70,4%)	1 (2,3%)	13,0	1,7 a 99,4	

4000 Hz					< 0,001

Suppression	26(59,1%)	40 (90,9%)	1,0		
No Suppression	18(40,9%)	4 (9,1%)	¥	5,5 a ¥	

6000 Hz					0,07

Suppression	23 (52,3%)	30 (68,2%)	1,0		
No Suppression	21 (47,7%)	14 (31,8%)	2,1	0,9 a 4,9	


OR = Odds ratio corresponding to the paired analysis.

p = Value corresponding to the McNemar Chi-Squared test.

In 1,000 and 6,000 Hz, tinnitus individuals tended to present less suppression than the controls (OR = 2.4; CI95%: 0.8 to 6.8; p = 0.09 and OR = 2.1; CI95%: 0.9 to 4.9). Notwithstanding, there was no statistically significant difference between both groups (p = 0.09 and p = 0.07, respectively).

As we can see by the OR and p values, the lack of DPOAE suppression in 1,500 Hz; 2,000 Hz; 3,000 Hz and 4,000 Hz was highly related to tinnitus.

## DISCUSSION

The literature is full of controversies about the function of the efferent auditory pathways and their true role on the human being auditory mechanisms.

Among the many functions previously assigned to these pathways, there would be the one responsible for triggering or maintaining tinnitus, through modulation alterations in organ or Corti^1^, ^9^, ^10^.

In patients with unilateral tinnitus, it has been shown less DPOAE suppression in the tinnitus side in relation to the contralateral side supression^11^, ^12^, thus suggesting an alteration in the inner workings of the efferent system in these cases. Notwithstanding, these results were not reproduced^14^ and, with a larger sample, less suppression was found in the contralateral side when compared to the tinnitus side^13^, thus pointing towards a disagreement in the literature about the dysfunction of the medial olivocochlear tract and tinnitus.

Since the auditory system is organized in a network pattern, with different communication points between the afferent and efferent systems^17^, the medial olivocochlear tract operates in a lateralized fashion, presenting higher suppressions in the right ear of right-handed individuals^7^, ^8^, it may be that such conflicting results in unilateral tinnitus patients may occur in function of comparing the data obtained in the right and left ears of the same individual, instead of using the control group.

Therefore, if an efferent dysfunction causes tinnitus in the left ear of a right-handed individual, the relationship between tinnitus and less suppression in this ear may be overestimated, because it is expected that the left side suppression be less in right-handed individuals. By the same token, if this same individual develops right side tinnitus, the relationship between tinnitus and less suppression may be underestimated, considering that suppression is usually higher on the right side of right-handed individuals. Having said that, we believe that the use of a control group is more adequate for this type of study, and not the contralateral ear of the same individual.

However, in order to use the control group, we had to be careful as to pair the tonal hearing thresholds and the DPOAE between the groups, since tinnitus patients may present values below these thresholds when compared to the control group, and this would lead to lower OAE suppression levels^18^, ^19^.

In a previous study, we had already compared the right ear DPOAE suppression in tinnitus patients to the right ear suppression in controls^20^. It is likely that due to the limited number of participants, we obtained only one significant relationship between the lack of DPOAE suppression and tinnitus in 4,000 Hz.

In the present investigation, with a larger sample, we observed an association between absent or little DPOAE contralateral suppression and the presence of tinnitus in all frequencies studied (OR>2.1). However, in 1,000 and 6,000 Hz, this association did not reach statistical significance level, suggesting that a greater increase in sample size could prove the correlation between tinnitus and efferent function alteration in all frequencies.

It is certain that the ideal research protocol for efferent hearing pathway functions is yet to be designed, and this is mainly due to the number of variables that need to be controlled. Thus, the results interpretation should be always based on the parameters used, since they may influence the responses without any clear clinical relation for that. In the specific case of this study, we believe that the asymmetrical functioning of the medial olivocochlear tract may alter the analysis of the results and this is a variable that has to be controlled.

## CONCLUSIONS

Despite the role of the efferent auditory pathways on the genesis and maintenance of tinnitus is still unclear, we showed that tinnitus patients, included in this investigation, presented an alteration in the medial olivocochlear tract function, seen by the lower DPOAE suppression in this group when compared to the group of patients without hearing complaints.
